# Supplementary material for: Recurrent predictive coding models for associative memory employing covariance learning
Source: PLoS Comput Biol. 2023 Apr 14;19(4):e1010719. doi: 10.1371/journal.pcbi.1010719 (PMC10132551; doi:10.1371/journal.pcbi.1010719)
Supplement: S1 Appendix — (PDF) [file pcbi.1010719.s001.pdf]

Supporting information for recurrent predictive coding models for associative memory employing covariance learning

Mufeng Tang[1], Tommaso Salvatori[2], Beren Millidge[1], Yuhang Song[1,2], Thomas Lukasiewicz[3,2], Rafal Bogacz[1*],

1 MRC Brain Network Dynamics Unit, University of Oxford, UK
2 Department of Computer Science, University of Oxford, UK
3 Institute of Logic and Computation, TU Wien, Austria

* rafal.bogacz@ndcn.ox.ac.uk

S1 Appendix. Here we provide the details of derivation of Eq 27, showing that all three models perform the same retrieval at convergence. At convergence, the retrieval dynamics of the explicit PCN satisfy:

$$S^{-1}(\mathbf{x} - \bar{\mathbf{x}}) = 0 \tag{35}$$

This equation can be written into its block matrix form:

$$\begin{bmatrix} S_{kk} & S_{km} \\ S_{mk} & S_{mm} \end{bmatrix}^{-1} \begin{bmatrix} \mathbf{x}_k - \bar{\mathbf{x}}_k \\ \mathbf{x}_m - \bar{\mathbf{x}}_m \end{bmatrix} = 0 \tag{36}$$

The inverse of S can thus be written as:

$$\begin{bmatrix} S_{kk} & S_{km} \\ S_{mk} & S_{mm} \end{bmatrix}^{-1} = \begin{bmatrix} Q^{-1} & -Q^{-1}S_{km}S_{mm}^{-1} \\ -S_{mm}^{-1}S_{km}^T Q^{-1} & S_{mm}^{-1} + S_{mm}^{-1}S_{km}^T Q^{-1} S_{km} S_{mm}^{-1} \end{bmatrix} \tag{37}$$

where Q is called the Schur complement of S_{mm} in S [1,2] and $Q = S_{kk} - S_{km}S_{mm}^{-1}S_{km}^T$. Since only $\mathbf{x}_k$ is relaxed during retrieval, we get:

$$Q^{-1}(\mathbf{x}_k - \bar{\mathbf{x}}_k) - Q^{-1}S_{km}S_{mm}^{-1}(\mathbf{x}_m - \bar{\mathbf{x}}_m) = 0 \tag{38}$$

which immediately gives us the retrieval dynamics in Eq 27.

Now we show that the retrieval of the implicit/dendritic models also follows Eq 27 at the convergence of inference. Notice that the sufficient and necessary condition for the convergence is $\boldsymbol{\varepsilon} = \mathbf{x} - W\mathbf{x} - \boldsymbol{\nu} = 0$. Splitting it into blocks corresponding to $\mathbf{x}_k$ and $\mathbf{x}_m$ this becomes:

$$\begin{bmatrix} W_{kk} & W_{km} \\ W_{mk} & W_{mm} \end{bmatrix} \begin{bmatrix} \mathbf{x}_k \\ \mathbf{x}_m \end{bmatrix} + \begin{bmatrix} \boldsymbol{\nu}_k \\ \boldsymbol{\nu}_m \end{bmatrix} = \begin{bmatrix} \mathbf{x}_k \\ \mathbf{x}_m \end{bmatrix} \tag{39}$$

Since we only relax the top k corrupted entries in $\mathbf{x}$, we have:

$$W_{kk}\mathbf{x}_k + W_{km}\mathbf{x}_m + \boldsymbol{\nu}_k = \mathbf{x}_k \Rightarrow (I_{kk} - W_{kk})\mathbf{x}_k = W_{km}\mathbf{x}_m + \boldsymbol{\nu}_k \tag{40}$$

We now investigate the parameter values in the above equation. Notice that we assumed the convergence of learning at the time of retrieval, which gives us Eqs 13 and 14. By splitting Eq 13 into blocks we have:

$$\boldsymbol{\nu}_k = (I_{kk} - W_{kk})\bar{\mathbf{x}}_k - W_{km}\bar{\mathbf{x}}_m \tag{41}$$

Substituting the $\boldsymbol{\nu}_k$ in Eq 40 with this relationship we get:

$$W_{km}(\mathbf{x}_m - \bar{\mathbf{x}}_m) = (I_{kk} - W_{kk})(\mathbf{x}_k - \bar{\mathbf{x}}_k) \tag{42}$$

Notice that Eq 14 connects the value of W and S, which can also be written into the block-matrix form:

$$\left(\begin{bmatrix} W_{kk} & W_{km} \\ W_{mk} & W_{mm} \end{bmatrix} \begin{bmatrix} S_{kk} & S_{km} \\ S_{mk} & S_{mm} \end{bmatrix} - \begin{bmatrix} S_{kk} & S_{km} \\ S_{mk} & S_{mm} \end{bmatrix} \right) \Big|_{diag=0} = 0 \tag{43}$$

which gives us two useful relationships:

$$W_{kk}S_{km} + W_{km}S_{mm} = S_{km} \Rightarrow W_{km} = (I_{kk} - W_{kk})S_{km}S_{mm}^{-1} \tag{44}$$

$$(W_{kk}S_{kk} + W_{km}S_{mk} - S_{kk})|_{diag=0} = 0 \tag{45}$$

Substituting the expression of W_{km} in terms of W_{kk} into Eq 42 we have:

$$(I_{kk} - W_{kk})S_{km}S_{mm}^{-1}(\mathbf{x}_m - \bar{\mathbf{x}}_m) = (I_{kk} - W_{kk})(\mathbf{x}_k - \bar{\mathbf{x}}_k) \tag{46}$$

The above expression is already close to Eq 27 which we seek to prove, i.e., we could obtain Eq 27 by cancelling $I_{kk} - W_{kk}$ on both sides of Eq 46, hence we will now show that $I_{kk} - W_{kk}$ is invertible. To do it we first observe that according to Eq 45, $W_{kk}S_{kk} + W_{km}S_{mk} - S_{kk}$ is a diagonal matrix, which we call D. Therefore we have:

$$\begin{aligned} D &= (I_{kk} - W_{kk})S_{kk} - W_{km}S_{mk} \\ &= (I_{kk} - W_{kk})S_{kk} - (I_{kk} - W_{kk})S_{km}S_{mm}^{-1}S_{mk} \\ &= (I_{kk} - W_{kk})(S_{kk} - S_{km}S_{mm}^{-1}S_{mk}) \\ &= (I_{kk} - W_{kk})Q \end{aligned} \tag{47}$$

Since Q is the Schur complement of S_{mm} in S, a sample covariance matrix that we assume to be positive definite, it is also positive definite and thus invertible [2]. Therefore $I_{kk} - W_{kk} = DQ^{-1}$. Notice that since W_{kk} has all its diagonal elements equal to 0, the matrix DQ^{-1} must have 1's on its diagonal, which prevents the diagonal matrix D from having 0's on its diagonal (for $\left(DQ^{-1}\right)_{ii} = D_{ii}\left(Q^{-1}\right)_{ii} = 1$, D_{ii} cannot be 0). Therefore, D is also invertible, which makes $I_{kk} - W_{kk}$ an invertible matrix as well. This enables us to "cancel out" the $I_{kk} - W_{kk}$ on both sides of Eq 46 and establish the equivalence to the retrieval of explicit covPCN (Eq 27).

References

1. Haynsworth EV. On the Schur Complement. Basel Univ (Switzerland) Mathematics Inst; 1968.
2. Boyd S, Vandenberghe L. Convex optimization. Cambridge university press; 2004.
