# Supplementary figures and images for: Recurrent predictive coding models for associative memory employing covariance learning
Source: PLoS Comput Biol. 2023 Apr 14;19(4):e1010719. doi: 10.1371/journal.pcbi.1010719 (PMC10132551; doi:10.1371/journal.pcbi.1010719)

A

1e-2 Retrieval MSE across different  $N$ s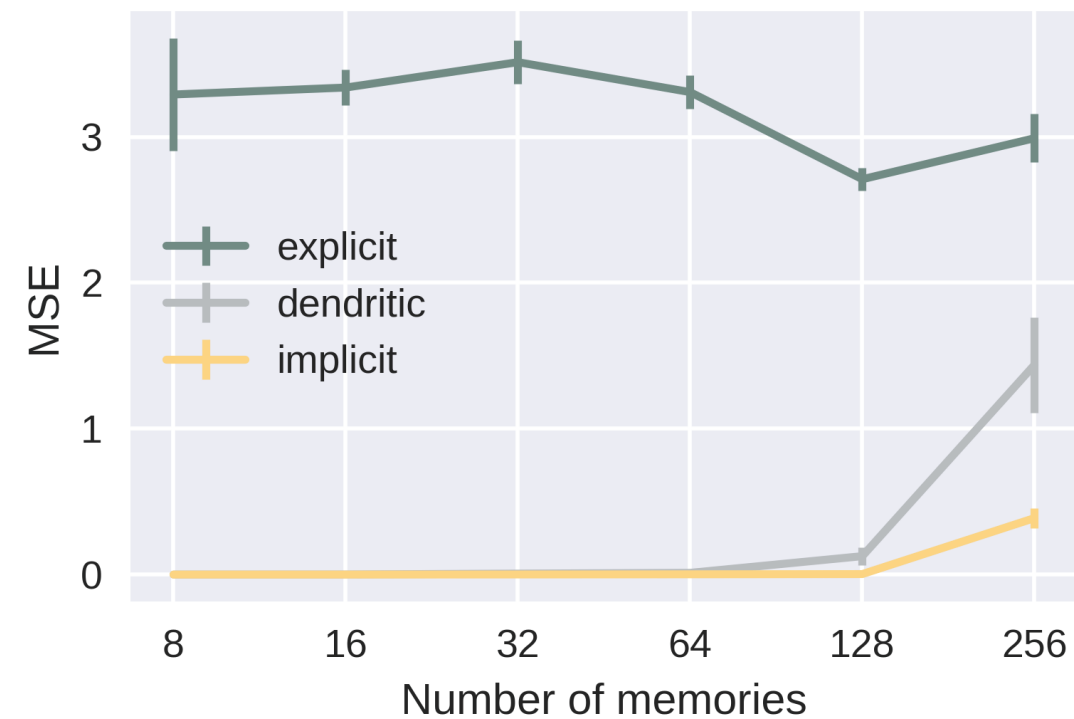

B

1e-3 Retrieval MSE across different  $N$ s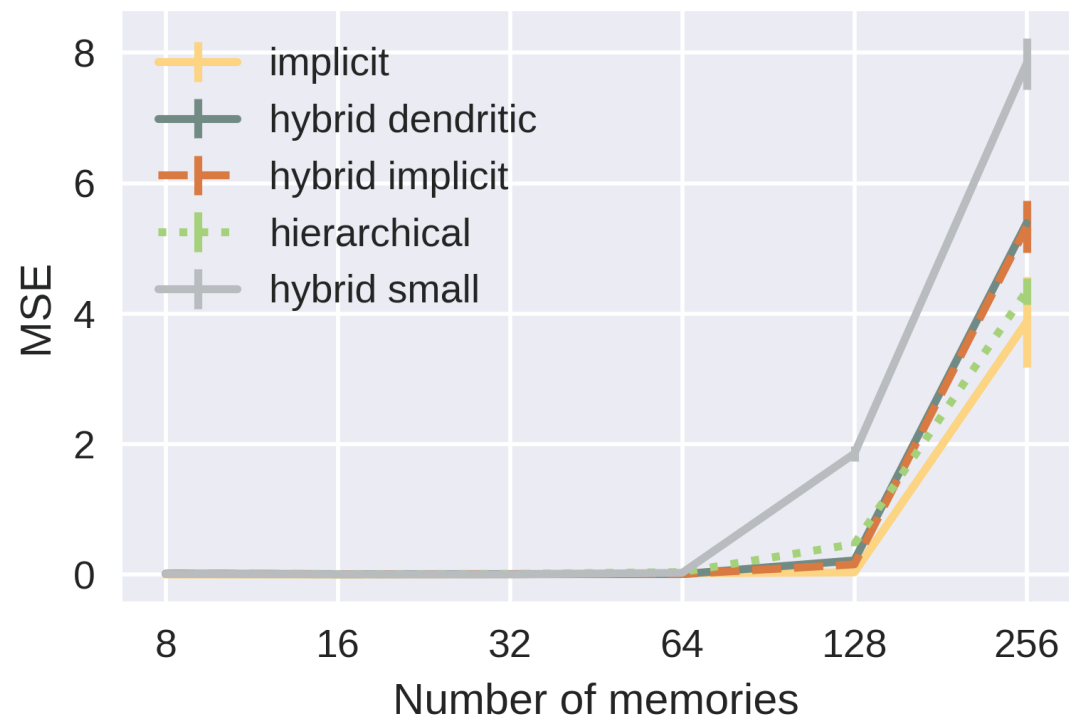

Supplement: S1 Fig — A: Retrieval MSEs of the single-layer models across multiple Ns. B: Retrieval MSEs of the multi-layer models across multiple Ns. (PDF) [file pcbi.1010719.s002.pdf]

A

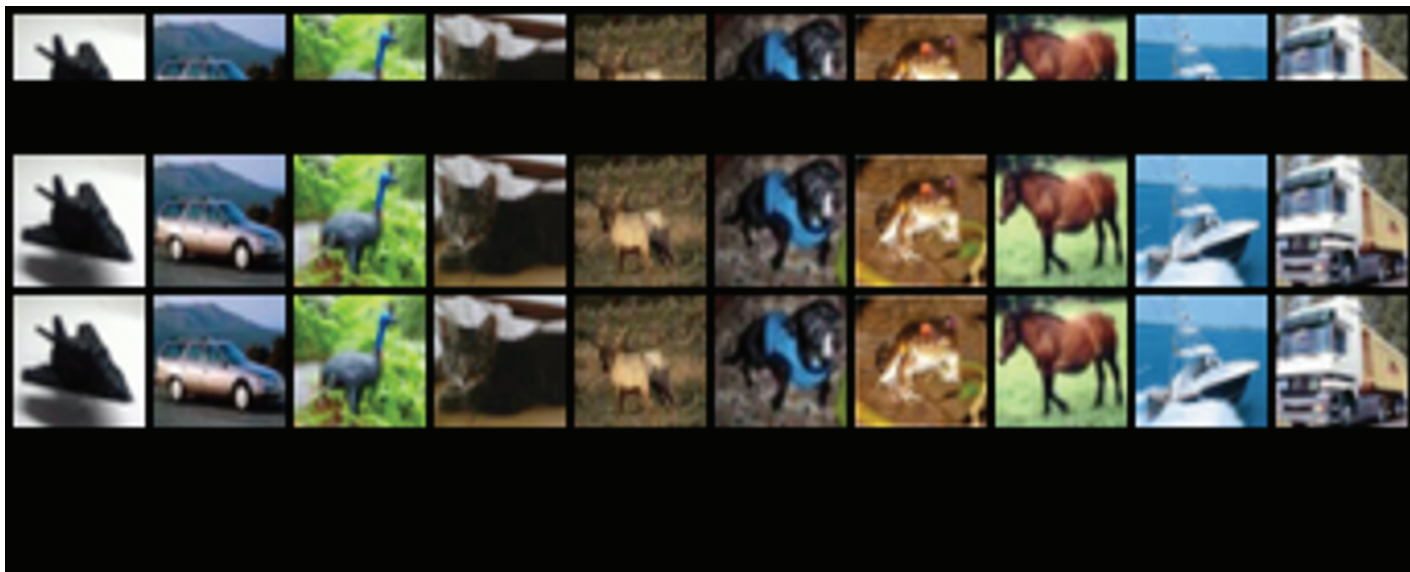

B

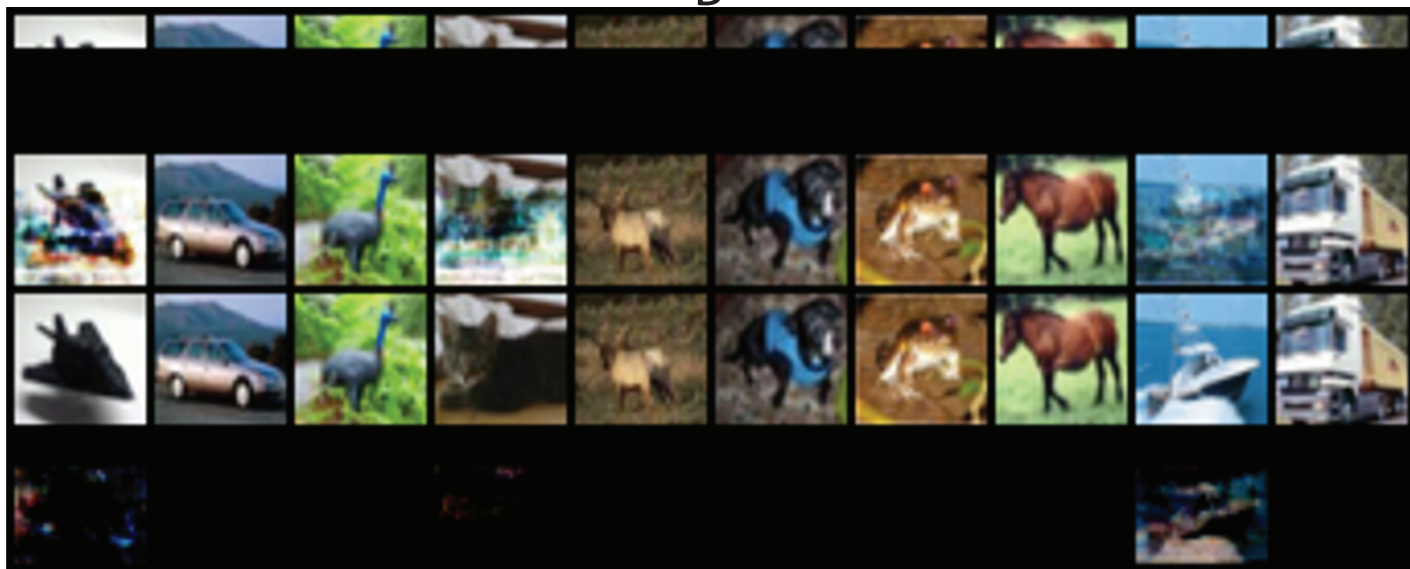

C

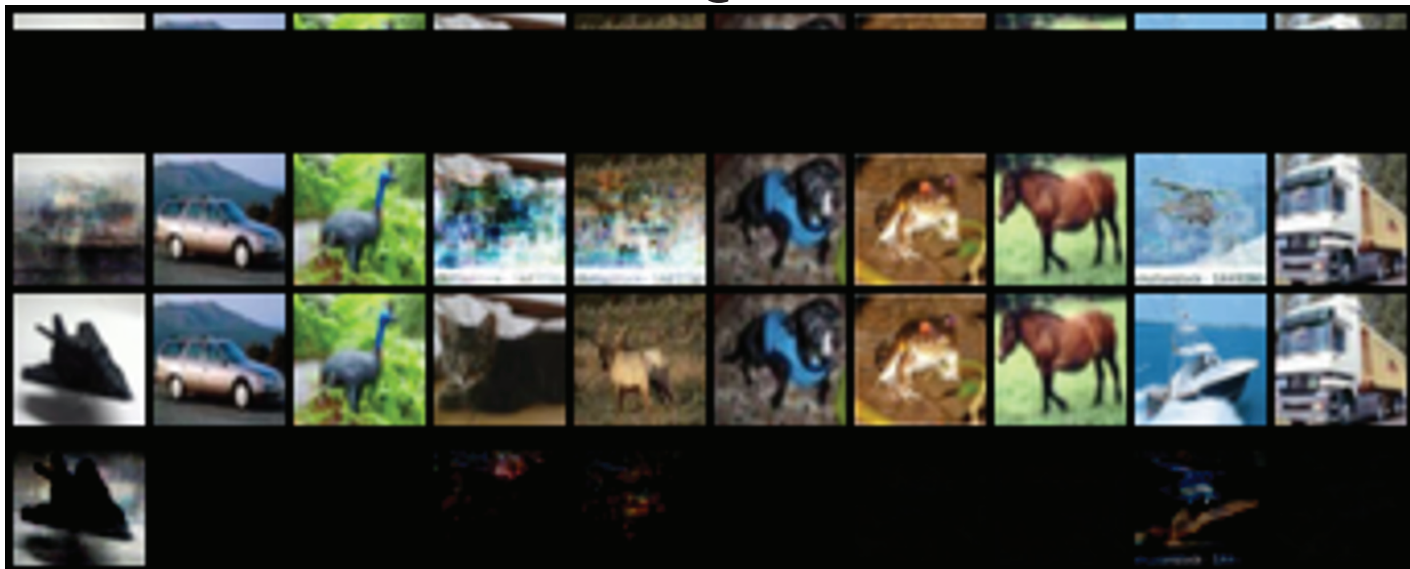

Supplement: S2 Fig — We also performed some preliminary experiments with the dendritic model on colored CIFAR10 images, and show some examples here. Particularly, we found that when presenting the network a cue consisting of 1/2 (panel A), 1/4 (panel B) and 1/8 (panel C) of the original pixels, the model has successfully recovered, respectively and on average, 89, 79, and 49 of the original memories in the experiments using colored CIFAR10. (PDF) [file pcbi.1010719.s003.pdf]
