# Supplementary material for: Recurrent predictive coding models for associative memory employing covariance learning
Source: PLoS Comput Biol. 2023 Apr 14;19(4):e1010719. doi: 10.1371/journal.pcbi.1010719 (PMC10132551; doi:10.1371/journal.pcbi.1010719)
Supplement: S1 Table — For all sample size of memories N, we use a batch size of N/8. For the inference iterations with the multi-layer models, the first number 400 is the number of inference iterations during training and within each training iteration. The second number 100000 is the number of inference iterations used to retrieve the original patterns. (PDF) [file pcbi.1010719.s004.pdf]

| model | corruption | $\alpha$ | learning iters | $\beta$ | inference iters |
|---|---|---|---|---|---|
| explicit | cover half | 1.00E-05 | 800 | 1.00E-01 | 5000 |
| implicit/dendritic | cover half | 1.00E-04 | 800 | 1.00E-01 | 50000 |
| implicit/nonlinear implicit | noise (var=0.1) | 1.00E-04 | 800 | 1.00E-03 | 20000 |
| nonlinear implicit | cover half | 1.00E-04 | 800 | 1.00E-01 | 50000 |
| hybrid/hierarchical | cover half | 1.00E-03 | 200 | 1.00E-02 | 400/100000 |
