# Supplementary material for: Recurrent predictive coding models for associative memory employing covariance learning
Source: PLoS Comput Biol. 2023 Apr 14;19(4):e1010719. doi: 10.1371/journal.pcbi.1010719 (PMC10132551; doi:10.1371/journal.pcbi.1010719)
Supplement: S2 Table — For all sample sizes of memories N, we use a batch size of N/8. For the inference iterations with the multi-layer models, the first number 400 is the number of inference iterations during training and within each training iteration. The second number 100000 is the number of inference iterations used to retrieve the original patterns. (PDF) [file pcbi.1010719.s005.pdf]

model	corruption	α	learning iters	β	inference iters
explicit	cover half	1.00E-05	1000	1.00E-01	5000
implicit/dendritic	cover half	2.00E-04	1000	2.00E-02	50000
hybrid/hierarchical	cover half	1.00E-03	200	1.00E-02	400/100000
