# Supplementary material for: Recurrent predictive coding models for associative memory employing covariance learning
Source: PLoS Comput Biol. 2023 Apr 14;19(4):e1010719. doi: 10.1371/journal.pcbi.1010719 (PMC10132551; doi:10.1371/journal.pcbi.1010719)
Supplement: Attachment — Submitted filename: Review plos paper by Bogacz.pdf [file pcbi.1010719.s006.pdf]

Summary
The authors extend previous models [23-25] developed in the context of Predictive Coding (PC) theories to account for Associative Memory (AM) tasks. The authors introduce a number of models (intrinsic, dendritic, hierarchical, nonlinear) and quantify their performance on traditional pattern retrieval (AM) tasks. They clearly expose the details of their framework and carry out a careful theoretical analysis of their results. The results are overall interesting and well-explained.

Assessment
The exposition is clear and the work appears solid, I haven't found any quantitative mistake in the authors analysis. However, I found the paper misleading in a few aspects. Specifically, the setup of the paper doesn't appear to point to the work carried out by the authors. I will explain this more in details below. Therefore I would suggest a careful assessment of whether the way the paper is presented is appropriate for the findings of the paper. Unfortunately, the predictive coding literature is very diverse and has several threads, I believe this prompts a more careful introduction on a number of aspects.

Major conceptual points
There are two major issues, on which I would like to draw the attention of the authors.
- First, the abstract and introduction appear to cast the work as reconciling the predictive and associative nature of hippocampal coding. However, such tension in the hippocampal literature arise between results linking temporal prediction to hippocampal phenomena (replay, preplay, time cells, the work on successor representation that the authors cite...). The associative versus spatial nature of the hippocampus is a debate which goes back to Eichenbaum and others, and has recently seen interesting literature arising in the context of predictive models to reconcile these two functions (spatio-temporal and associative) but all the predictive work, at least to the best of my knowledge, is in the temporal domain. The authors, on the contrary, focus their work on a predictive model which is in the spatial domain as they eventually point out in lines 695-702. This can be extremely misleading for the average reader and should at least be discussed clearly in the introduction. I actually wonder whether the entire dichotomy set up by the authors in the abstract and introduction (AM versus PC for hippocampal functions) is well justified in light of what they do. Temporal predictive coding is very different from spatial predictive coding, which indeed is not too far from pattern completion. Are then the results of the authors surprising along this avenue?
- Second, the authors exploit their framework to model AM. The traditional reference model for AM in the hippocampal literature is the Hopfield model where stable point attractors are "written" in the dynamic/connectivity of the network by means of a specific connectivity matrix. The commonality between the Hopfield model and other AM attractor models is that the memories are stable point attractors. The authors lack to discuss whether their network really "writes" stable point attractors in the dynamic/connectivity of the network till the introduction of their nonlinear model. Only there, unless I missed a previous discussion, they state that their framework actually defines an hyperplane of fixed points. Only with the introduction of the non-linearity these hyperplane is compressed to a set of points. If this is correct, please do correct me if I am wrong, then this point ought to be discussed upfront as the set of models that they introduce lacks to reproduce this basic feature of AM networks: the full stability of the fixed

points. Mathematically it would be a positive definite hessian around each fixed point. As the author nicely build their work up to the nonlinear model, maybe that should have more emphasis and be part of the narrative at an earlier stage.

While I like the paper, the models and the results, I would like the authors to help me clarify the points above.

Minor points

- Lines 83-87: it is not clear whether these two contributions are a step forward or not from 23-25. I would suggest rewriting these two major contributions, at least for the first one it sounds like the explicit model had already achieved that for the way it is written.

- The corruption expriments of Fig 3A are not very satisfactory. A plot comparing the retrieval efficacy of the different algorithms as a function of corruption (maybe as a function of the std of added gaussian noise to each entry of the figure) would be interesting.

- The text is lenghty and not fully clear in all places. For example the comment in lines 472-478 doesn't seem to be based on an important result (the lines are not significantly separated, at least to the eye) and the comment seems to be a better fit for the discussion.

- Line 516: Fig 3C: linear implicit cov PCN learn line attractor in the 2-dimensional space. Can you expland this discussion and move it up in the narrative. See conceptual point above. Maybe the very definition of associative memory should be introduced, and compared with Hopfield case right away.

- Lines 695-702: The discrimination between spatial and temporal PC should come upfront becuase this is a major discrimanation and potential limitation of the proposed framework.

Typos
51: biologicaly -> biological
81: italic for hierarchical cov PCN.
