# Supplementary material for: Recurrent predictive coding models for associative memory employing covariance learning
Source: PLoS Comput Biol. 2023 Apr 14;19(4):e1010719. doi: 10.1371/journal.pcbi.1010719 (PMC10132551; doi:10.1371/journal.pcbi.1010719)
Supplement: Attachment — Submitted filename: review-response-new.docx [file pcbi.1010719.s007.docx]

# Response to the comments of the Editor and Reviewers

We thank the Editor and the Reviewers for their careful reading of our manuscript and for providing very useful comments which helped us to improve the manuscript. We have addressed all the comments in the revised version of the manuscript, and the modified parts of the manuscript are highlighted with red ink in the manuscript file. We restate each of the comments below (in blue italics), and describe how we addressed it. We also add line numbers to each of our solutions (where appropriate) so that the Reviewers can find the revisions more easily.

## Reviewer #1:

*I enjoyed reading this compelling and thoughtful treatment of different predictive coding algorithms — and how they may be implemented by the hippocampus. I thought your explanation of this sort of predictive coding (and the underlying schemes) was clear and accessible. However, I think you need to make it easier for the reader to understand why your scheme works. Furthermore, it might help both you and the reader understand the various implementations of PCN you have considered in the larger context of predictive coding in neuroscience and engineering. I have suggested how you could do this by unpacking and qualifying some of your key points.*

***Major points***
 *It would be helpful for the reader to explain why your simplifications work — and take the opportunity to explain what kind of predictive coding you are using to illustrate your ideas. I would recommend the following at the end of the introduction:

“In summary, we describe a series of simpler and more biologically plausible implementations of predictive coding that we frame in terms of associative memory and hippocampal function in the brain. To unpack the basic ideas, we will focus on a simple kind of predictive coding, in which we ignore dynamics and temporal prediction. Furthermore, we will restrict our initial analyses to linear systems, under standard parametric assumptions (i.e., Gaussian assumptions about random effects). In this setting, a memory is simply the ability of the PCN to recognize the most likely cause of a particular pattern of inputs (e.g., an image). We are not addressing episodic memory – of the sort sometimes associated with hippocampal function — rather, we are focusing on how statistical regularities in a series of inputs are learned and then used to predict the underlying cause of a corrupted or random input.

In terms of inference and learning, predictive coding can be regarded as a special class of optimization schemes — that maximize the marginal likelihood of generative models via the minimization of variational free energy — under Gaussian or parametric assumptions. These Gaussian assumptions mean that the requisite free energy — gradients that drive inference and learning — can be expressed as prediction errors, which is the definitive aspect of predictive coding. The key thing that we bring to the table is a reparameterization of the prediction errors, which leads to simplified forms of a free energy. Crucially, the simplified forms of free energy we consider all share the same minima or fixed points; thereby leading to the same inference and learning. Practically, this allows us to drop certain terms from the gradients (i.e., prediction errors), leading to more robust convergence to free energy minima and, crucially, affording more biologically plausible implementation."*

We added these paragraphs into the Introduction, with some minor modifications to fit them into the context. **[Line 94-123]**
 *When you first mention the energy or objective function on line 100, please refer explicitly to “(variational) free energy” (as an upper bound on marginal likelihood). Your work has moved beyond the use of arbitrary energy functions found in neural networks and machine learning and is using an energy which bounds model evidence or marginal likelihood.*

Done **[Line 128]** *On line 131, please include a footnote or sentence along the following lines:

“Although we refer to these estimates maximum likelihood estimates (MLE), in more general formulations they would correspond to maximum a posteriori (MAP) estimates. However, because we have not placed any prior constraints on the implicit generative model implied by equation (1), the MLE and MAP become the same thing."*

Done **[Line 165-169]** *The section on nonlinear predictive coding networks and the learning of individual attractors, is interesting but could be removed. I suggest this because the particular functional form of the nonlinearity in (33) is not motivated in any principled way. And the connection with attractors needs to be developed more rigorously. In other words, I think you need to think about this more carefully before committing to the literature.

Alternatively, you could qualify this section by including the following at the end:

“In (33) we have introduced an ad hoc nonlinearity into the definition of the free energy – as motivated by previous heuristics in neural networks and machine learning. One way to understand these heuristics more formally is to appreciate that the free energy is a statement about the underlying generative model that generates inputs (e.g., images) from some latent causes. For example, the generative model implied by the free energy in equation (1) is a general linear model, which explains why we can understand its inversion with PC in terms of regression coefficients and the standard linear algebra found in parametric statistics.

Adding nonlinearities to generative models of this sort usually leads to sparse coding schemes of the kind found in independent component analysis (Olshausen and Field, 1996). This suggests an interesting relationship between sparse coding, predictive coding and the properties evinced by the numerical studies above. Clearly, nonlinearities also speak to more expressive generative models of the kind with we can leverage in the hierarchical and hybrid schemes demonstrated above. In future work, we will revisit the above schemes starting from the underlying generative model, from which the free energy functional and their gradients (i.e., prediction errors) can then be derived."*

We agree with the reviewer that this section needs to be better motivated. However, instead of adding the paragraphs above to the end, we adopted the two main motivations suggested by the reviewer, namely 1) heuristics in neural networks and machine learning and 2) nonlinear models are potentially more expressive, and added them to the beginning of the section where we first introduced the nonlinear models. We hope this will make the motivations for the nonlinear models even clearer before we formally introduce them. We also think the connection to sparse coding is very interesting, and added it the literature review at the end of the paper. **[Line 540-547, 652-655]**

*On line 582, you provide a brief history of PC. This history makes it appear that you do not know the predictive coding literature or developments outside machine learning. I recommend you speed read: Does predictive coding have a future? | Nature Neuroscience. And include the following to summarize PC:

“Linear predictive coding was first introduced for the compression of timeseries data in the 1950s (Elias, 1955). In the neurosciences, it was originally proposed to explain the efficient processing of visual inputs to the retina (Srinivasan et al., 1982) and subsequently used to model hierarchical processing in the visual cortex, with a special focus on extra-classical receptive field effects (Rao and Ballard, 1999). As noted above, predictive coding is a particular class of variational inversion schemes for hierarchical generative models under Gaussian or parametric assumptions (Friston, 2008). Under state space generative models, the corresponding scheme is known as a Kalman filter for linear models – and an extended Kalman filter for non-linear models (Rao, 1999). Usually, predictive coding deals with state space models and implicit predictions about dynamics it was later extended to account for a range of neuronal responses from…"

In light of the above, you might want to refer to the subsequent developments of predictive coding based upon the variational formulation. The work based upon references [23] and [24] means that your comment on line 720 may need to be revised. In other words, there is quite a large literature on predictive coding and temporal prediction and learning. For example: (Friston and Kiebel, 2009; Kiebel et al., 2009). There are some entertaining examples using birdsong; especially numerical illustrations of predictive coding that involve learning – or indeed recognizing who is singing.*

We added most of these references to our discussion. **[Line 631-639, 778-788]** *You may also want to include the following in your discussion of future directions:

“In our current work, we have focused on learning covariance or precision matrices inherent in a batch of training images. Hierarchical implementations of predictive coding often use more expressive generative models, in which the covariance or precisions at various hierarchical levels can change over time. This means that instead of learning the covariances, they are inferred; leading to the notion of dynamic precision weighting of prediction errors (Clark, 2013; Limanowski, 2017). This may be an interesting avenue to pursue; particularly, because it provides a model of attention from a neuroscience perspective – that may be related to the kinds of attention found in transformers and the like."*

We added this to our future directions. **[Line 796-804]**

*Minor points

Line 47, you might replace “dichotomy” with “dialectic"?*

We rewrote the Introduction and avoided to frame our work as to solve the dichotomy (based on reviewer 2’s comments). This word does not exist any more.
 *Line 144, I did not quite understand the phrase "corresponding training data point" perhaps it would be clearer to say:

“For example, if x~ is a corrupted or noisy data point, the equation above will drive it to its nearest point within the learned Gaussian density. One can see this from equation (5) in which the estimate is pulled towards the mean in proportion to the precision or inverse variance."*

Done **[Line 175-176]** *Line 166: did you mean to say: "which are not generated by sampling from a Gaussian distribution"?*

Yes, we changed it. **[Line 198]**
 *Line 268: please say "as a memory index".*

Done **[Line 299]**
 *Line 286: please say "a property never previously demonstrated with this model. We then derive an expression for a retrieved…"*

Done **[Line 318]**

 *For each figure (when appropriate) could you specify the number of latent states (i.e., d) used for the simulations. For example, you clearly used two dimensional causes for Figure 3. However, it is not clear how many states (i.e., the dimensionality) you used for the hierarchical and hybrid schemes. It would be easiest for the reader to have this information in each figure legend (as opposed to a table).*

For Figure 3 and 4, we added the number of dimensions used in each experiment to the figure captions, as all models are single-layer and thus the dimensions are the same as the number of pixels in the inputs. For Figure 5 we added the specific number of hidden dimensions to panel B for our experiments with grayscale CIFAR10 *Line 360, please replace “encode” with "encodes"*

Done

*In the legend to figure 4, please add “(please see main text for an explanation of the matrix M)”.*

Done
 *Line 474, please replace "functionalities" with "functionality"*

Done *Line 475: please replace "requires" with "require".*

Done
 *Line 498: please replace "negative energy function with "negative free energy function".*

This phrase no longer exists, after the modifications following the major points above.
 *Line 683: You might want to refer to the work of Jeffrey Gray, who spent much of his life theorising about prediction errors and the role of the hippocampus in learning (for example, learning and latent inhibition).*

We referred to Jeffrey Gray’s book “The Neuropsychology of Anxiety: An Enquiry into the Functions of the Septo-Hippocampal System” at the point where we discuss the hippocampal role in predicting sensory inputs. **[Line 740, reference 64]**
 *I hope that these suggestions help should any revision be required.


Clark, A., 2013. The many faces of precision (Replies to commentaries on "Whatever next? Neural prediction, situated agents, and the future of cognitive science"). Front Psychol 4, 270.
Elias, P., 1955. Predictive coding–I. IRE Transactions on Information Theory 1, 16–24.
Friston, K., 2008. Hierarchical models in the brain. PLoS Comput Biol 4, e1000211.
Friston, K., Kiebel, S., 2009. Predictive coding under the free-energy principle. Philosophical transactions of the Royal Society of London. Series B, Biological sciences 364, 1211-1221.
Kiebel, S.J., von Kriegstein, K., Daunizeau, J., Friston, K.J., 2009. Recognizing sequences of sequences. PLoS Comput Biol 5, e1000464.
Limanowski, J., 2017. (Dis-)Attending to the Body, in: Metzinger, T.K., Wiese, W. (Eds.), Philosophy and Predictive Processing. MIND Group, Frankfurt am Main.
Olshausen, B.A., Field, D.J., 1996. Emergence of simple-cell receptive field properties by learning a sparse code for natural images. Nature 381, 607-609.
Rao, R.P., 1999. An optimal estimation approach to visual perception and learning. Vision Res 39, 1963-1989.
Rao, R.P.N., Ballard, D.H., 1999. Predictive coding in the visual cortex: a functional interpretation of some extra-classical receptive-field effects. Nature Neuroscience 2, 79-87.
Srinivasan, M.V., Laughlin, S.B., Dubs, A., 1982. Predictive coding: a fresh view of inhibition in the retina. Proc R Soc Lond B Biol Sci 216, 427-459.*

## Reviewer #2

***Summary***

*The authors extend previous models [23-25] developed in the context of Predictive Coding (PC) theories to account for Associative Memory (AM) tasks. The authors introduce a number of models (intrinsic, dendritic, hierarchical, nonlinear) and quantify their performance on traditional pattern retrieval (AM) tasks. They clearly expose the details of their framework and carry out a careful theoretical analysis of their results. The results are overall interesting and well-explained.*

***Assessment***

*The exposition is clear and the work appears solid, I haven't found any quantitative mistake in the authors analysis. However, I found the paper misleading in a few aspects. Specifically, the setup of the paper doesn't appear to point to the work carried out by the authors. I will explain this more in details below. Therefore I would suggest a careful assessment of whether the way the paper is presented is appropriate for the findings of the paper. Unfortunately, the predictive coding literature is very diverse and has several threads, I believe this prompts a more careful introduction on a number of aspects.*

***Major conceptual points***

*There are two major issues, on which I would like to draw the attention of the authors.*

***- First****, the abstract and introduction appear to cast the work as reconciling the predictive and associative nature of hippocampal coding. However, such tension in the hippocampal literature arise between results linking temporal prediction to hippocampal phenomena (replay, preplay, time cells, the work on successor representation that the authors cite...). The associative versus spatial nature of the hippocampus is a debate which goes back to Eichenbaum and others, and has recently seen interesting literature arising in the context of predictive models to reconcile these two functions (spatio-temporal and associative) but all the predictive work, at least to the best of my knowledge, is in the temporal domain. The authors, on the contrary, focus their work on a predictive model which is in the spatial domain as they eventually point out in lines 695-702. This can be extremely misleading for the average reader and should at least be discussed clearly in the introduction. I actually wonder whether the entire dichotomy set up by the authors in the abstract and introduction (AM versus PC for hippocampal functions) is well justified in light of what they do. Temporal predictive coding is very different from spatial predictive coding, which indeed is not too far from pattern completion. Are then the results of the authors surprising along this avenue?*

We rewrote the whole Introduction to clarify at the beginning of the paper that the PC models discussed in our work considers only the spatial domain. We also removed the description about the dichotomy between PC and AM as the motivation/setup of our work. Instead, we motivate our work with the **exploration of implementing PC within recurrent networks that can be considered as models of AM**, which also extends and completes the hierarchical PC models proposed by Salvatori et al. (2021) as a detailed model implementation of the theories by Barron et al. (2020). We hope this modification clarifies the motivation of this work and connects the presentation to the findings of the paper. **[Line 9-122]**

***- Second****, the authors exploit their framework to model AM. The traditional reference model for AM in the hippocampal literature is the Hopfield model where stable point attractors are "written" in the dynamic/connectivity of the network by means of a specific connectivity matrix. The commonality between the Hopfield model and other AM attractor models is that the memories are stable point attractors. The authors lack to discuss whether their network really "writes" stable point attractors in the dynamic/connectivity of the network till the introduction of their nonlinear model. Only there, unless I missed a previous discussion, they state that their framework actually defines an hyperplane of fixed points. Only with the introduction of the non-linearity these hyperplane is compressed to a set of points. If this is correct, please do correct me if I am wrong, then this point ought to be discussed upfront as the set of models that they introduce lacks to reproduce this basic feature of AM networks: the full stability of the fixed points. Mathematically it would be a positive definite hessian around each fixed point. As the author nicely build their work up to the nonlinear model, maybe that should have more emphasis and be part of the narrative at an earlier stage. While I like the paper, the models and the results, I would like the authors to help me clarify the points above.*

In the summarizing paragraph of the Introduction we added descriptions that restrict the scope of AM discussed in our work: First, we added that we are not addressing episodic memory, which corresponds to the scope of PC (spatial instead of temporal) discussed in our paper; Second, we added that AM in our discussions is the ability of a model to predict missing or noisy parts of a memory input based on learned statistical regularities from a batch of inputs. This contrasts our models with attractor models such as Hopfield Networks. We also pre-empt in this paragraph that we will have some initial discussion about the attractor behavior of our models, although more rigorous investigations are indeed needed in future works to build up the connections to classical attractor networks. **[Line 92-122]**

***Minor points***

*- Lines 83-87: it is not clear whether these two contributions are a step forward or not from*

*23-25. I would suggest rewriting these two major contributions, at least for the first one it sounds like the explicit model had already achieved that for the way it is written.*

We removed these contributions and replaced them earlier in the Introduction with a research question we addressed in this work: “how can the recurrent hippocampal network employ PC to perform AM, in a biologically plausible and computationally stable manner that is also reconcilable with covariance learning?” **[Line 52-56]**

*- The corruption expriments of Fig 3A are not very satisfactory. A plot comparing the retrieval*

*efficacy of the different algorithms as a function of corruption (maybe as a function of the std of added gaussian noise to each entry of the figure) would be interesting.*

We added the numerical results on the 5x5 random patterns to this figure. Specifically, in Figure 3B we added a plot of the retrieval MSE as a function of number of masked pixels (masked by 0). Interestingly, thanks to the reviewer’s comments, we noticed that the explicit model already has a larger retrieval MSE than the implicit and dendritic models, although this performance gap is not visually observable on the retrieved patterns. We also select a set of random patterns with higher contrast to make the visual demonstration of the retrieval fidelity clearer and thus more convincing. **[Line 342-350]**

*- The text is lenghty and not fully clear in all places. For example the comment in lines 472-478 doesn't seem to be based on an important result (the lines are not significantly separated, at least to the eye) and the comment seems to be a better fit for the discussion.*

We agree that in this case the implicit model doesn’t really have significantly better performance than the hybrid model, and have changed the description to “the similar performance of the implicit and hybrid model doesn’t make the hierarchical structure in the hybrid model redundant”. However, to reduce the cross reference of results in the Discussion section and keep a clear structure of presentation, we decided to keep this comment immediately next to our result. We hope this clarifies the paper. We are also more than happy to edit and provide clarification, should there be any other specific parts of the paper that the reviewer thinks are also lengthy and unclear. **[Line 518-520]**

*- Line 516: Fig 3C: linear implicit cov PCN learn line attractor in the 2-dimensional space. Can you expland this discussion and move it up in the narrative. See conceptual point above. Maybe the very definition of associative memory should be introduced, and compared with Hopfield case right away.*

Done in Introduction; we also noted in the description of Fig 3C that the linear attractor points to a different type of AM than Hopfield Nets. **[Line 110-122]**

*- Lines 695-702: The discrimination between spatial and temporal PC should come upfront*

*becuase this is a major discrimanation and potential limitation of the proposed framework.*

Done in Introduction **[Line 104-107]**

*Typos*

*51: biologicaly -> biological*

*81: italic for hierarchical cov PCN.*

We rewrote the Introduction so these two typos no longer exist.
